# The CT-based attenuation index of peri-bowel adipose tissue can predict disease progression in inflammatory bowel disease patients

**DOI:** 10.1007/s00330-025-12079-x

**Published:** 2025-10-22

**Authors:** Jun Lu, Hui Xu, Jingxuan Zhang, Tianxin Cheng, Jing Zheng, Xinjun Han, Yuxin Wang, Xuxu Meng, Xiaoyang Li, Jiahui Jiang, Xue Dong, Zhenchang Wang, Zhenghan Yang, Lixue Xu

**Affiliations:** Department of Radiology, Beijing Friendship Hospital, Capital Medical University, Beijing, China

**Keywords:** Inflammatory bowel disease, Peri-bowel fat, Fat attenuation index, Disease progression, Risk stratification

## Abstract

**Objectives:**

Peri-bowel fat inflammation is a prominent feature of inflammatory bowel disease (IBD). The peri-bowel fat attenuation index (FAI) can capture fat inflammation on abdominal CT. This study aimed to investigate the prognostic value of the peri-bowel FAI in IBD patients.

**Materials and methods:**

Totally, 207 IBD patients were retrospectively collected. Regions of interest were placed at 5 different locations, namely, mesenteric side (MS) and opposite side of MS (OMS) around the most severe bowel lesion, spaces around the normal bowel wall (Nor), retroperitoneal space (RS), and subcutaneous area. The Kaplan–Meier curves were plotted. The prognostic value of the peri-bowel FAI was evaluated by multivariable Cox regression models.

**Results:**

High peri-bowel FAI values of MS and OMS were predictors of disease progression and correlated strongly with each other (r = 0.840, *p* < 0.001), while the FAI of Nor and RS were not. Therefore, peri-bowel FAI of MS was used as a representative biomarker for the prediction of IBD disease progression (HR = 1.161 [1.110–1.215], *p* < 0.001) with an optimum cutoff of 25.1 HU, which was confirmed in the subgroup analysis with different disease subtypes. With the addition of the peri-bowel FAI to the current noninvasive risk prediction model, the AUC increased from 0.706 (0.638–0.767) to 0.864 (0.810–0.90) with integrated discrimination improvement (IDI = 0.293 [0.229–0.356], *p* < 0.001) and net reclassification improvement (NRI = 1.053 [0.821–1.284], *p* < 0.001).

**Conclusion:**

The peri-bowel FAI is promising for IBD disease progression prediction and risk stratification by quantifying peri-bowel fat inflammation. High peri-bowel FAI values are an independent indicator of increased IBD disease progression and could guide early targeted prevention and intensive therapy.

**Key Points:**

***Questions*** *The peri-bowel fat attenuation index (FAI) helps detect peri-bowel fat inflammation noninvasively, but its importance for risk stratification and prediction of clinical outcomes remains unknown.*

***Findings*** *The peri-bowel FAI was an independent predictor of inflammatory bowel disease (IBD) disease progression with an optimum cutoff of 25.1 HU.*

***Clinical relevance*** *The peri-bowel FAI is a promising biomarker for contributing to the identification of so-called high-risk patients with uncontrolled inflammation, who might be candidates for more intensive treatment for addressing underlying inflammation at early stages and ultimately improve long-term prognosis.*

**Graphical Abstract:**

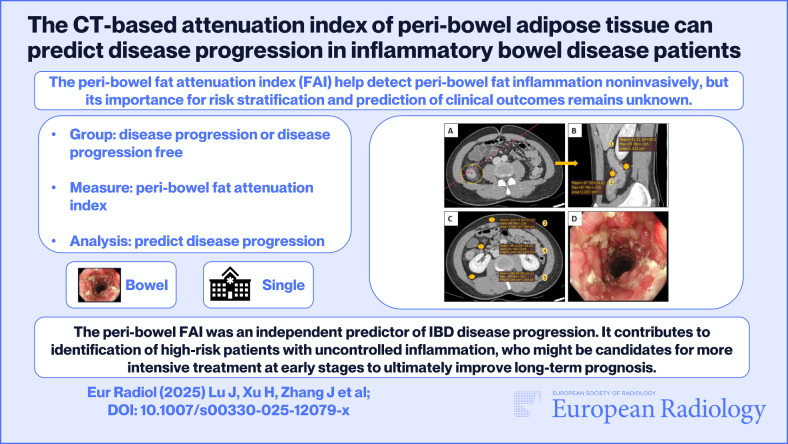

## Introduction

Inflammatory bowel disease (IBD) is characterized by chronic, progressive immune-mediated inflammation, and mainly includes ulcerative colitis (UC) and Crohn’s disease (CD) [1]. With the in-depth understanding of the pathogenesis of IBD and the development and application of a variety of therapeutic drugs, the ultimate therapeutic targets emphasize the importance of looking beyond symptoms and addressing underlying inflammation, minimizing disease activity at early stages and limiting progression [2–4]. Recognition of the importance of managing both symptoms and inflammation in IBD patients has contributed to the consideration of “treat-to-target” management approach, which helps limit progression and improve patient-centered outcomes [5]. This approach involves setting an appropriate goal, selecting initial therapy according to the risk of disease progression and monitoring disease progression [6, 7].

At present, endoscopy, which is correlated with disease activity and used for monitoring the treatment efficacy, is the mainstay of IBD evaluation. However, invasive examination methods, rigorous bowel preparation, expensive fees, and general reluctance to repeated endoscopy might represent pivotal hurdles to endoscopy. Actually, there is a low utilization rate of colonoscopy (from 26.6% to 63.2%) for the assessment of therapeutic efficacy in real-world clinical practice according to a multicenter prospective adult research cohort with IBD [8]. Several clinical laboratory indicators such as C-reactive protein (CRP), erythrocyte sedimentation rate (ESR) and fecal calprotectin (FC) are noninvasive risk factors for disease progression and can facilitate the monitoring of disease activity to some extent [9, 10], but these biomarkers are not currently recognized as the ultimate targets in the Selecting Therapeutic Targets in Inflammatory Bowel Disease (STRIDE) recommendations based on a tight control strategy [4, 5]. Computed tomography enterography (CTE) and magnetic resonance enterography (MRE) have become routine cross-sectional imaging tools to assess disease activity and disease-related complications in IBD [2, 11]. Accurate visual assessment results during CTE and MRE examinations require adherence to relevant bowel preparation guidelines. But the peri-bowel fat inflammation induced by bowel disease can be observed and quantified without special bowel preparation. Bowel wall inflammation and inflammation-induced barrier dysfunction are typical features of IBD lesions, leading to an increase in bowel permeability. The consequent translocation of viable bacteria to visceral adipose tissue (VAT) could affect the local release of inflammatory mediators [12]. VAT is not only a storage organ but also an endocrine organ, which is a possible source of pro-inflammatory substances as described in previous studies [13, 14]. Previous studies have confirmed that the VAT is closely related to disease severity and treatment response [15]. The peri-bowel adipose tissue (PBAT) surrounding the bowel lesion, which is the pioneer of VAT exposed to enterobacteria, became a physiologically active fat compartment with the adipocyte hyperplasia and the production of pro-inflammatory substance [12, 16]. This excessive immune response is reflected by dynamic changes in the balance between water and lipid content, which can be evaluated by a novel imaging biomarker—the peri-bowel fat attenuation index (FAI)—that captures these inflammation-induced changes in peri-bowel fat attenuation based on conventional abdominal computed tomography (CT).

The peri-bowel FAI can easily detect peri-bowel fat inflammation, but its importance for risk stratification and prediction of clinical outcomes remains unknown. We postulated that the peri-bowel FAI could predict disease progression by quantifying peri-bowel fat inflammation, independent of current noninvasive risk factors such as laboratory indicators, thus achieving the goals of identifying high-risk IBD patients who might benefit from intensive therapeutic strategies, controlling underlying inflammation at early stages and improving long-term outcomes.

## Materials and methods

### Patients and study design

**Fig. 1:**
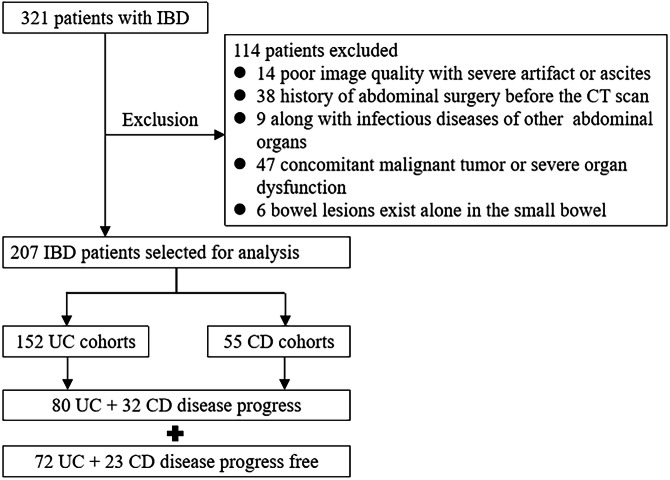
The study design and patient selection

This retrospective study was approved by the institutional review board, and the requirement for informed consent was waived (2024-P2-031-01). This study enrolled patients consecutively with a confirmed diagnosis of CD or UC from August 2017 to March 2023. The inclusion criteria were as follows: (1) adults with a definite diagnosis of IBD based on clinical manifestations, endoscopy, imaging analysis, and histological criteria; (2) available abdominal CT images within 1 week before or after endoscopic examination; (3) available complete clinical data within 1 week before or after abdominal CT from electronic medical records; and (4) a follow-up of at least 1 year. The exclusion criteria were as follows: (1) poor image quality with severe artifacts; (2) a history of abdominal surgery; (3) along with infectious diseases of other abdominal organs; (4) concomitant malignant tumor or severe organ dysfunction; and (5) bowel lesions exist alone in the small bowel beyond the scope of ileocolonoscopy. If there were multiple CT scans, the first CT scan was utilized. The study design and patient selection criteria are shown in Fig. 1.

### Primary outcome

The primary outcome was IBD disease progression, which was defined as IBD-related enterectomy or colectomy, IBD-related hospitalization or emergency room visit, initiation of steroids, change of IBD medication due to uncontrolled inflammation, dose or frequency increase of IBD medication due to ongoing inflammation. Information related to disease progression was manually retrieved from electronic medical records. The concept of IBD disease progression has been proposed in the past, specifically devoted to identifying risk factors for disease progression [17, 18].

### CT-based fat attenuation index (FAI) measurements

#### Target bowel selection

All the abdominal CT images were obtained according to clinically standardized protocols from four CT scanners. The specific details of the conventional CT scan protocols are shown in Supplementary Table 1. The contrast-enhanced CT images would be performed at the portal venous phase (about 65–70 s) using iodine contrast media of 350–370 mg/mL with a total dose of 80–100 mL injected at a rate of 3–5 mL/s. The bowel evaluated by colonoscopy was divided into 5 parts: the terminal ileum, right-sided colon involving the ascending colon and hepatic flexure, transverse colon involving the splenic flexure, left-sided colon involving the descending colon, sigmoid colon and rectum. A previous study confirmed that the most severe endoscopic lesion was associated with the worst disease outcome [19]. Therefore, the specific part of the most severe bowel lesion of each patient was selected as the target bowel, defined by the endoscopic findings according to the scoring systems of the Ulcerative Colitis Endoscopic Index of Severity (UCEIS) for UC and the Simple Endoscopic Score for CD [20, 21]. Then, the corresponding CT images of the most severe bowel lesion were subsequently analyzed.

### FAI measurement

All the regions of interest (ROIs) were independently placed on CT images by two radiologists (with 5 and 8 years of experience in abdominal CT) using conventional Picture Archiving and Communication Systems (PACS). They were blinded to the patients’ clinical data and outcomes. The mean value of the FAI first recorded by two radiologists was used for analysis. To ensure the inter-reader agreement, one of the radiologists repeated the measurements 1 month later.

**Fig. 2:**
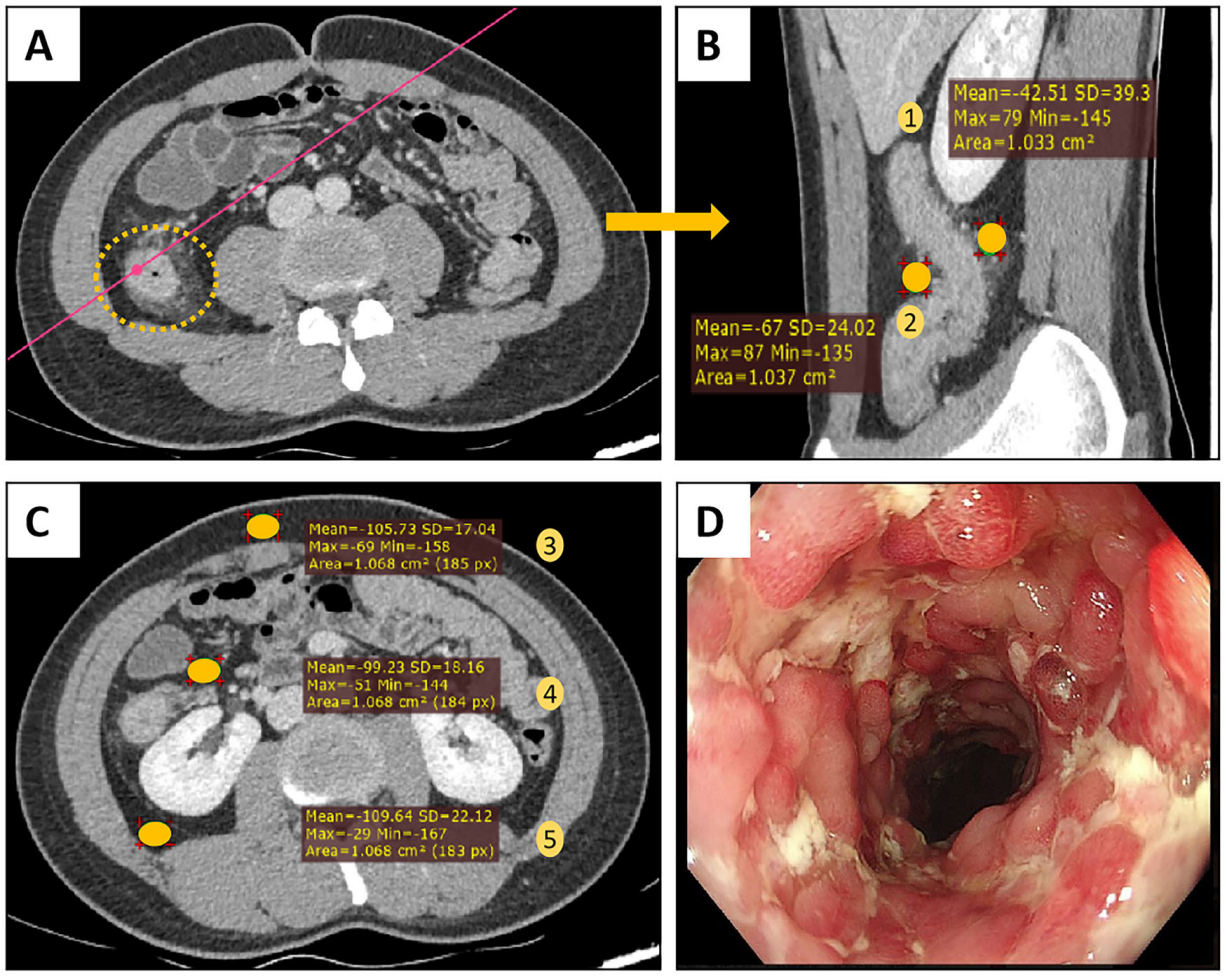
Quantitative assessment of the fat attenuation index (FAI) at 5 different locations. Abdominal CT image of a 22-year-old Crohn’s disease patient whose most severe bowel lesion was located at the ascending colon. **A** The yellow circle indicated the bowel lesion. **B** The oblique sagittal section is reconstructed based on the pink line. **C** Transverse section of the third lumbar vertebra (L3). **D** Endoscopic image of the bowel lesion. The numbers 1–5 represent 5 different locations, namely, the mesenteric side (MS) and the opposite side of MS (OMS) around the most severe bowel lesion, the subcutaneous area, the space around the normal bowel wall (Nor) and the retroperitoneal space (RS), respectively

The FAI was measured by placing ROIs at 5 different locations including the mesenteric side (MS) and opposite side of MS (OMS) of the most severe bowel lesion, space around the normal bowel wall (Nor), retroperitoneal space (RS) and subcutaneous area in the portal venous phase abdominal CT images (Fig. 2). For each location, ROIs with an area of 1–2 cm^2^ were manually placed avoiding the visible mesenteric vessels and lymph glands.

After that, the fat attenuation index was normalized by calculating the difference value with the fat attenuation of MS, OMS, Nor and RS minus that of the subcutaneous area, respectively.

### Statistical analysis

The intra- and inter-observer reliability of the FAI was assessed by calculating the intraclass correlation coefficient (ICC). The Kolmogorov-Smirnov test was used to assess the normal distribution of continuous variables. Continuous variables are reported as the means ± SD or median (interquartile range (IQR)) according to whether data were normally distributed or not. Categorical variables are presented as numbers and percentages. Continuous variables were compared using Student’s *t*-test or Mann–Whitney U Test. The chi-square test was used to evaluate distributions of categorical variables. The correlations between continuous variables were determined using Pearson’s correlation or Spearman’s correlation, as appropriate.

The optimum cutoff of the peri-bowel FAI for distinguishing IBD disease progression was defined by the value that maximized Youden’s J statistic on the time-dependent receiver operating characteristic (ROC) curve. The Kaplan–Meier curves were plotted based on the different risk groups with high or low peri-bowel FAI values. The prognostic value of the peri-bowel FAI for IBD disease progression was evaluated by multivariable Cox regression models.

By adding the peri-bowel FAI to a baseline model consisting of traditional noninvasive risk factors, improvements in model performance, discrimination and risk classification were assessed by (1) comparing the ROC curves by the Delong method; and (2) calculating the integrated discrimination improvement (IDI) and net reclassification improvement (NRI) indices. The prediction performance and prognostic value of the peri-bowel FAI for IBD disease progression were also assessed in subgroup analyses according to the different disease phenotypes (CD or UC).

Statistical analysis was performed with the IBM SPSS software (version 22.0), MedCalc (version 19.6) and R software (version 4.3.0). All tests were two-sided, and *p* < 0.05 was considered statistically significant.

## Results

### Patient characteristics

A total of 207 patients in the active phase were enrolled, with 152 UC and 55 CD patients (Table 1). The target lesions were located at the terminal ileum, right-sided colon, transverse colon, left-sided colon, sigmoid colon and rectum with number of 23 (11.11%), 40 (19.32%), 23 (11.11%), 37 (17.88%) and 84 (40.58%), respectively. During the follow-up of all the IBD patients, 112 were confirmed disease progression and 95 confirmed disease-progression-free. The details of disease progression are shown in Supplementary Table 2. Only laboratory data were significantly statistically different, and other characteristics showed no statistical difference between the IBD disease progression and disease-progression-free groups (Table 2).

**Table 1:** Patient characteristics at the baseline, stratified by disease phenotype

Characteristics	Total (*n* = 207)	Ulcerative colitis (*n* = 152)	Crohn’s disease (*n* = 55)
Age (years)	48 (34–59)	51 (39–59)	30 (27–52)
Sex, *n* (%)			
Female	86 (41.5)	74 (48.7)	12 (21.8)
Male	121 (58.5)	78 (51.3)	43 (78.2)
BMI (kg/m^2^)	22.10 (19.44–23.88)	22.36 (20.83–24.67)	19.49 (17.80–22.84)
Disease subtype, *n* (%)			
E1	--	11 (7.2)	--
E2	--	43 (28.3)	--
E3	--	98 (64.5)	--
L1 (terminal ileum)	--	--	14 (25.5)
L2 (colon)	--	--	17 (30.9)
L3 (ileocolon)	--	--	19 (34.5)
L4 (upper)	--	--	0 (0.0)
L1 + L4	--	--	1 (1.8)
L2 + L4	--	--	3 (5.5)
L3 + L4	--	--	1 (1.8)
Endoscopic scores	--	5 (4–6)^b^	6 (4–7)^c^
Disease duration (months)	36 (12–120)	36 (9–138)	30 (12–96)
Medication type, *n* (%)			
ASA only	101 (48.8)	83 (54.6)	18 (32.7)
Steroid	37 (17.9)	32 (21.1)	5 (9.1)
Immunomodulator^a^	4 (1.9)	2 (1.3)	2 (3.6)
Biologic	65 (31.4)	35 (23.0)	30 (54.6)
Laboratory data			
ESR (mm/h)	17.00 (6.00–41.00)	17.50 (7.00–44.75)	11.00 (5.00–34.00)
CRP (mg/L)	4.98 (2.06–26.97)	4.80 (2.12–26.77)	4.98 (1.15–45.03)
Neutrophil count (10^9^/L)	3.70 (2.53–4.89)	4.02 (2.77–5.22)	3.16 (1.94–4.30)
Albumin (g/L)	36.40 (32.50–39.50)	34.14 ± 5.60	36.40 (32.80–39.60)
Clinical outcome, *n* (%)			
Disease progression	112 (54.1)	80 (52.6)	32 (58.2)
Disease progression-free	95 (45.9)	72 (47.4)	23 (41.8)
Follow-up time (months)	18.0 (12.0–22.0)	18.0 (12.0–22.8)	16.0 (12.0–20.0)

Continuous variables are described with mean ± standard deviation or median (interquartile range), as appropriate. Categorical variables are described with number (percentage)

*BMI* body mass index, *E1* proctosigmoiditis, *E2* left-side colitis, *E3* pancolitis, *ASA* 5-aminosalicylic acid, *ESR* erythrocyte sedimentation rate, *CRP* C-reactive protein

^a^ Including patients of immunomodulator + ASA (*n* = 1) in Crohn’s disease

^b^ Ulcerative Colitis Endoscopic Index of Severity (UCEIS)

^C^ Simple Endoscopic Score

**Table 2:** Comparison of baseline characteristics and risk factors between disease-progression-free and disease progression groups

Characteristics	Total (*n* = 207)		Statistic value	*p*-value
	Disease progression-free (*n* = 95)	Disease progression (*n* = 112)		
Age (years)	48 (34–59)	38 (31–62)	−1.084	0.278
Sex, *n* (%)			1.600	0.206
Female	35 (36.8)	51 (45.5)		
Male	60 (63.2)	61 (54.5)		
BMI (kg/m^2^)	22.10 (19.44–23.88)	21.67 (19.72–24.91)	−0.028	0.978
Disease duration (months)	36 (12–120)	36 (10–96)	−0.223	0.824
Smoke status, *n* (%)			1.209	0.546
Never	72 (75.8)	79 (70.5)		
Previous	17 (17.9)	27 (24.1)		
Current	6 (6.3)	6 (5.4)		
Laboratory data				
ESR (mm/h)	17.00 (6.00–41.00)	27.00 (13.00–43.50)	2.811	0.005
CRP (mg/L)	4.98 (2.06–26.97)	17.90 (4.46–37.80)	2.469	0.014
Neutrophil count (10^9^/L)	3.70 (2.53–4.89)	4.68 (3.39–6.84)	3.402	0.001
Albumin (g/L)	36.40 (32.50–39.50)	34.40 (29.95–37.77)	−2.549	0.011

Continuous variables are described with mean ± standard deviation or median (interquartile range), as appropriate. Categorical variables are described with number (percentage)

*BMI* body mass index, *ESR* erythrocyte sedimentation rate, *CRP* C-reactive protein

### The optimal cutoff value of the peri-bowel FAI

The peri-bowel FAI values showed high repeatability with all ICC ≥ 0.871 (Supplementary Table 3). The peri-bowel FAI values of the MS and OMS were significantly different between the disease progress and disease-progression-free groups (*p* < 0.001), but the FAI values of the Nor and RS were not (*p* = 0.117 and 0.129, respectively).

The FAI values of the MS and OMS were closely correlated with the endoscopic scores (r = 0.540 and 0.461, respectively, *p* < 0.001). The peri-bowel FAI of MS was associated strongly with the peri-bowel FAI of OMS (r = 0.840, *p* < 0.001). In view of the statistical collinearity between peri-bowel FAI measurements around the most severe bowel lesion, the analysis was restricted to the FAI of MS. Therefore, the study population was dichotomized into high versus low peri-bowel fat attenuation groups based on an optimal cutoff of 25.1 HU (calculated at a median follow-up of 18 months) with Youden’s J index of 0.556, a sensitivity of 83.50% and a specificity of 72.12%.

### Risk stratification of clinical outcomes with peri-bowel FAI

**Fig. 3:**
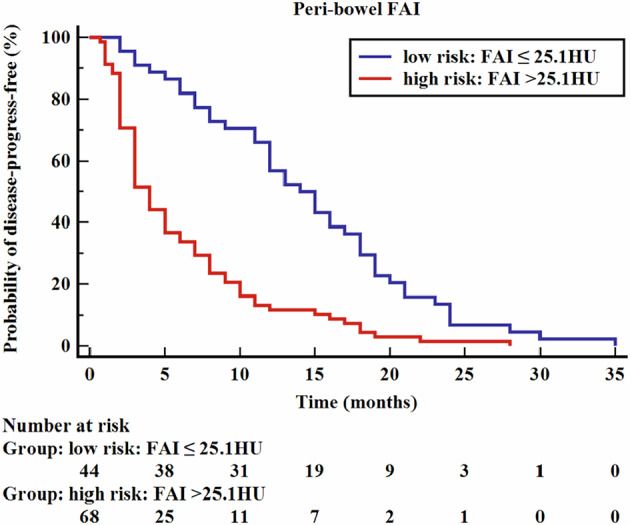
Kaplan–Meier curve for the risk of IBD disease progression with high versus low peri-bowel FAI (log-rank *p* < 0.001). IBD, inflammatory bowel disease; FAI, fat attenuation index

With the Kaplan–Meier survival analysis, peri-bowel FAI values of 25.1 HU or higher were associated with increased risk of IBD disease progression (log-rank *p* < 0.001) (Fig. 3). The median disease-progression-free survival of the high-risk groups was significantly shorter than that of the low-risk groups (4 months vs. 14 months, *p* < 0.001).

**Fig. 4:**
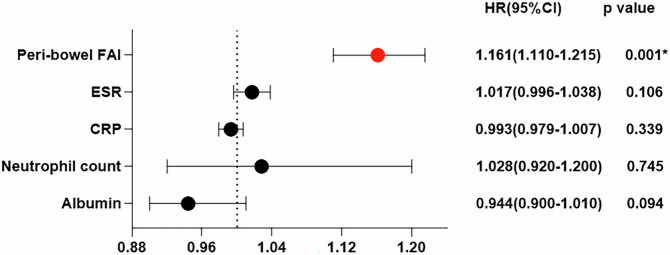
Forest plot of Cox regression analysis with noninvasive risk factors (the peri-bowel FAI and laboratory data: ESR, CRP, neutrophil count and albumin) in IBD patients. The peri-bowel FAI was an only independent predictor of IBD disease progression. FAI, fat attenuation index; IBD, inflammatory bowel disease; ESR, erythrocyte sedimentation rate; CRP, C-reactive protein

A multivariable Cox regression model was constructed to evaluate the relationships between the peri-bowel FAI and IBD disease progression. Because the disease activity at the baseline was associated with the peri-bowel FAI measurement, the disease activity was excluded from the Cox regression analysis. The peri-bowel FAI and significant laboratory data were included in the Cox regression analysis. In the final model, only peri-bowel FAI was strongly associated with IBD disease progression (hazard ratio (HR), 1.161; 95% confidence interval (CI): 1.110–1.215; *p* < 0.001), as shown in Fig. 4.

### Incremental prediction value of the peri-bowel FAI

**Fig. 5:**
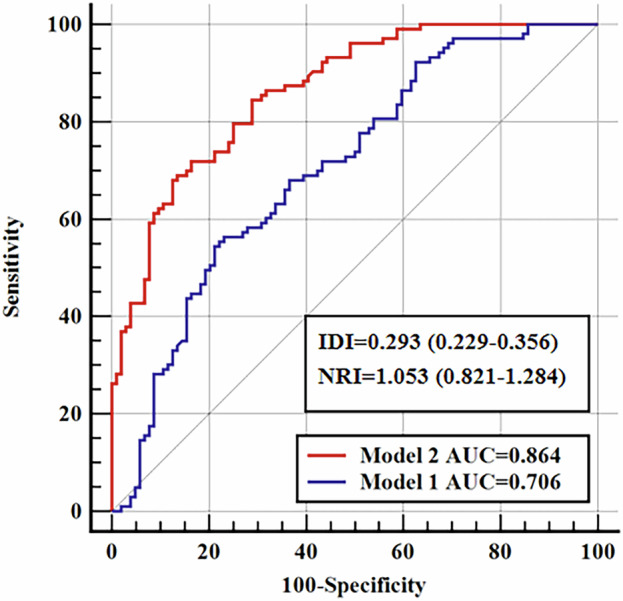
Incremental prognostic value of the peri-bowel FAI for risk stratification. Time-dependent ROC curves (at 18 months) for the discrimination and risk classification of IBD disease progression. Model 1 represents the current noninvasive risk assessment and consists of the CRP, ESR, neutrophil count and albumin. Model 2 incorporates the peri-bowel FAI values into Model 1. ROC, receiver operating characteristic; IBD, inflammatory bowel disease; CRP, C-reactive protein; ESR, erythrocyte sedimentation rate; IDI, integrated discrimination improvement; NRI, net reclassification improvement

The inclusion of the peri-bowel FAI values significantly improved the discriminating value of the risk prediction model, which included CRP, ESR, neutrophil count and albumin for IBD disease progression. With the addition of the peri-bowel FAI to the risk prediction model, the AUC increased from 0.706 (95% CI: 0.638–0.767) to 0.864 (95% CI: 0.810–0.908) (∆AUC = 0.158; *p* < 0.001) (Fig. 5) at a median follow-up of 18 months. Furthermore, the peri-bowel FAI significantly improved IBD disease progression risk stratification, contributing to substantial improvements in integrated discrimination improvement (IDI = 0.293 (0.229–0.356), *p* < 0.001) and net reclassification improvement (NRI = 1.053 (0.821–1.284), *p* < 0.001).

### Subgroup analysis by disease phenotypes

To test whether the relationships between the peri-bowel FAI and disease progression differ between UC and CD cohorts. The study population was classified into UC and CD cohorts. Similar results were found in the UC and CD cohorts compared to the overall study population.

With the peri-bowel FAI cutoff value of 25.1 HU for UC and 28.5 HU for CD, respectively, the median disease-progression-free survival of the high-risk groups was significantly shorter than that of the low-risk groups (UC: 4 months vs. 15 months, log-rank *p* < 0.001; CD: 3 months vs. 13 months, log-rank *p* = 0.001) (Supplementary Fig. 1).

With the multivariable Cox regression analysis, the peri-bowel FAI was the only independent prognostic factor for disease progression in both the UC and CD cohorts. An obvious association was observed between the peri-bowel FAI and disease progression in UC patients (HR: 1.191 [95% CI: 1.121–1.266], *p* < 0.001), whereas the relationship was similar for those with CD patients (HR: 1.126 [95% CI: 1.034–1.227], *p* = 0.006), as shown in Supplementary Fig. 2.

With the addition of the peri-bowel FAI to the laboratory data-based risk prediction model, the model performance, discrimination and risk classification improved in the UC cohort (∆AUC_UC_ = 0.207, *p* < 0.001; IDI = 0.356 (0.278–0.433), *p* < 0.001; NRI = 1.026 (0.753–1.299), *p* < 0.001) and in the CD cohort (∆AUC_CD_ = 0.054, *p* < 0.001; IDI = 0.143 (0.046–0.240), *p* = 0.003; NRI = 0.624 (0.131–1.118), *p* = 0.013), as shown in Supplementary Fig. 3. Besides, there was no statistical difference between ∆AUC_UC_ and ∆AUC_CD_ (Z = 1.676, *p* = 0.094).

## Discussion

The findings of our study showed that the noninvasive imaging biomarker peri-bowel FAI can predict disease progression. By quantifying the peri-bowel fat inflammation, the peri-bowel FAI can be used for risk stratification. Integrating the peri-bowel FAI into the current laboratory risk factor prediction model will contribute to the identification of individuals at risk of future disease progression, and will flag so-called high-risk patients with uncontrolled inflammation, who might be candidates for more intensive treatment for addressing underlying inflammation at early stages.

Current noninvasive risk stratification relies on traditional risk factors, whereas laboratory data such as CRP levels reflect general body conditions and might be inconsistent with the severity of the bowel illness [22]. In addition, the serum CRP is not specific enough for detecting IBD-related inflammation [23]. Addressing the underlying inflammation is a crucial element in the “treat to target” management approach [24]. Therefore, noninvasive detection of the peri-bowel fat inflammatory risk could guide more timely deployment of intensive measures in initial treatment strategies [2, 4].

The impaired barrier function and increased bowel permeability are prominent features of IBD lesions. This structural disruption facilitates the translocation of gut bacteria into peri-bowel adipose tissue, which has been implicated in the pathogenesis and disease progression of IBD [25, 26]. As the pioneer of VAT exposed to translocated gut bacteria, the peri-bowel adipose tissue undergoes significant immune cell infiltration and upregulated expression of pro-inflammatory mediators [12, 27]. Peri-bowel adipose tissue, functioning as a major secretory organ for pro-inflammatory cytokines and pathogenic adipokines, has been mechanistically linked to the pathogenesis of IBD. It is expected to be a novel therapeutic target for modulating the inflammatory microenvironment in IBD [28].

In our study, this excessive immune response was quantitatively evaluated by the peri-bowel FAI. The information captured by the peri-bowel FAI is independent of laboratory risk factors such as CRP, ESR and neutrophil count. Previous studies have shown that the volume and mass of the VAT were closely correlated with therapeutic efficacy and disease progression [18, 29]. Another study [30] concluded that the fat attenuation at the third lumbar vertebra was related to the short-term prognosis of UC patients. However, the inflammation in VAT is not distributed evenly based on visual assessment. We observed that peri-bowel fat inflammation around the bowel lesion was more obvious than that far away from the bowel wall. Finally, our study confirmed that a high peri-bowel FAI was associated with a significantly high risk of disease progression, but high FAI values of fat around the normal bowel wall and retroperitoneal space were not. In addition, the peri-bowel FAI in our study was normalized by fat attenuation of the subcutaneous area to exclude the interference of tube voltage and individual variation as much as possible [31, 32]. In our study, 88 (42.51%) IBD patients underwent abdominal CT scans without corresponding bowel preparation. The evaluation of bowel wall CT signs might be influenced, but the peri-bowel FAI could be measured accurately. Thus, we were confident that the peri-bowel FAI was potentially in daily clinical practice for evaluation of peri-bowel fat inflammation and identification of high-risk IBD patients.

Although the study population was a mixture of UC and CD patients, the conclusions still showed remarkable robustness across different subgroups. In subgroup analysis according to the different disease phenotypes, a cutoff for the peri-bowel FAI of 25.1 HU or higher was a strong predictor of UC disease progression, and 28.5 HU or higher was also a strong predictor of CD disease progression. With the addition of the peri-bowel FAI to the laboratory data-based risk prediction model, the model performances in both CD and UC subgroups were closely aligned with the entire cohort analysis. More importantly, the peri-bowel FAI was an only independent prognostic factor for disease progression, and the association between peri-bowel FAI and disease progression showed no statistical difference between the CD and UC subgroups. In a 2013 early study, Zulian et al [33] reported that CD adipocytes were more densely colonized with gut bacteria, which in turn promoted adipocyte proliferation. These prior conclusions inspired researchers to thoroughly explore the potential role of adipocytes in IBD pathogenesis and to confirm whether there exist differences in the pathogenesis mechanisms between UC and CD cohorts.

Our study confirmed that the peri-bowel FAI could predict disease progression and identify high-risk IBD patients who might benefit from more intensive therapeutic strategies at early stages. A clear unmet need still exists to identify patients who remain at risk after achieving short-term goals in line with the “treat to target” management approach due to a repeated relapsing-remitting clinical course of IBD. In the future, more clinical studies should be carried out to confirm whether the peri-bowel FAI is a long-awaited noninvasive biomarker that will guide personalized therapeutic strategies in IBD patients.

This study has certain limitations. Firstly, this was a retrospective and single-center study. Therefore, this conclusion should be confirmed in a prospective multicenter study in the future. Second, several factors that may influence the clinical outcomes analyzed in this study, such as disease location and distribution in CD, were not fully accounted for. We are looking forward to avoiding this selection bias in future studies. Besides, the manually placed ROIs in our study partially reflected the inflammation of the PBAT and were limited by the subjective selection of regions of interest in spite of a considerable repeatability. We are looking forward to identifying and segmenting three-dimensional PBAT via a deep learning algorithm to obtain valuable predictors more accurately and efficiently in the future.

## Conclusion

In summary, the peri-bowel FAI, a novel imaging biomarker when measured from the mesenteric side around the most severe bowel lesion, was an independent predictor of IBD disease progression with an optimum cutoff of 25.1 HU or higher. In addition, integration of the peri-bowel FAI into the current laboratory risk factor prediction model for identifying high-risk patients, the prediction performance improved and showed robustness across different disease phenotypes. The peri-bowel FAI is a promising noninvasive risk-stratification biomarker and will contribute to the identification of individuals at risk of uncontrolled inflammation who might be candidates for more intensive treatment and ultimately improve long-term prognosis.

## Supplementary information

ELECTRONIC SUPPLEMENTARY MATERIAL

## Abbreviations

CD: Crohn’s disease
CT: Computed tomography
FAI: Fat attenuation index
IBD: Inflammatory bowel disease
MS: Mesenteric side of the most severe bowel lesion
Nor: Space around the normal bowel wall
OMS: Opposite side of MS of the most severe bowel lesion
PBAT: Peri-bowel adipose tissue
RS: Retroperitoneal space
UC: Ulcerative colitis
VAT: Visceral adipose tissue
