# Supplementary material for: The CT-based attenuation index of peri-bowel adipose tissue can predict disease progression in inflammatory bowel disease patients
Source: Eur Radiol. 2025 Oct 22;36(4):2832–41. doi: 10.1007/s00330-025-12079-x (PMC13035559; doi:10.1007/s00330-025-12079-x)
Supplement: Supplementary file 1 — ELECTRONIC SUPPLEMENTARY MATERIAL [file 330_2025_12079_MOESM1_ESM.pdf]

# The CT-based attenuation index of peri-bowel adipose tissue can predict disease progression in inflammatory bowel disease patients

# ELECTRONIC SUPPLEMENTARY MATERIAL

Supplementary Table 1 The scanner parameters of the four computed tomography scanners

| CT scanners | GE Optima CT660 | Philips Brilliance iCT 256 | GE Revolution CT | Canon Aquilion one |
|---|---|---|---|---|
| Name | CT 1 | CT2 | CT3 | CT4 |
| Scanner parameters | | | | |
| Tube voltage, KVp | 120 | 120 | 120 | 120 |
| Tube current, mA | 80-500 | 60-290 | 10-490 | 50-150 |
| Slice thickness, mm | 1.25 | 1.5 | 1.25 | 1.0 |
| Scan pitch ratio | 1.375:1 | 0.914:1 | 0.992:1 | 0.813:1 |
| Reconstruction diameter,mm | 376 | 406 | 350 | 404 |
| Spacing between slices, mm | 1.25 | 1.5 | 1.25 | 1.0 |

Supplementary Table 2 The different disease progression subtype recorded per patient.

| Disease progression subtypes | patient, n (%) |
|---|---|
| Total | 112(100) |
| IBD-related colectomy | 6(5.4) |
| IBD-related hospitalization or emergency room visit[a] | 36(32.1) |
| Initiation of steroids[b] | 4(3.6) |
| Change of IBD medication[c] | 28(25.0) |
| Dose or frequency increase of IBD medication | 38(33.9) |

IBD, inflammatory bowel disease.

[a]Excluding those involved colectomy.

[b]Excluding steroids prescribed during hospitalization.

[c]Excluding steroids.

Supplementary Table 3 The intra- and interclass correlation coefficient of FAI

| FAI | ICC(95%CI) Radiologist 1 time 1 vs time 2 | ICC(95%CI) Radiologist 1 vs Radiologist 2 |
|---|---|---|
| FAI-MS | 0.927(0.833, 0.965) | 0.901(0.862, 0.934) |
| FAI-OMS | 0.893(0.834, 0.920) | 0.871(0.825, 0.911) |
| FAI-Nor | 0.845(0.762, 0.898) | 0.828(0.737, 0.889) |
| FAI-RS | 0.838(0.778, 0.881) | 0.788(0.749, 0.862) |

The interval between time 1 and time 2 is one month. ICC, intra- and interclass correlation coefficient; FAI, fat attenuation index. The MS, OMS, Nor and RS refer to 4 different locations, including mesenteric side (MS) and opposite side of MS (OMS) around the most severe bowel lesion, space around the normal bowel wall (Nor) and retroperitoneal space (RS), respectively.

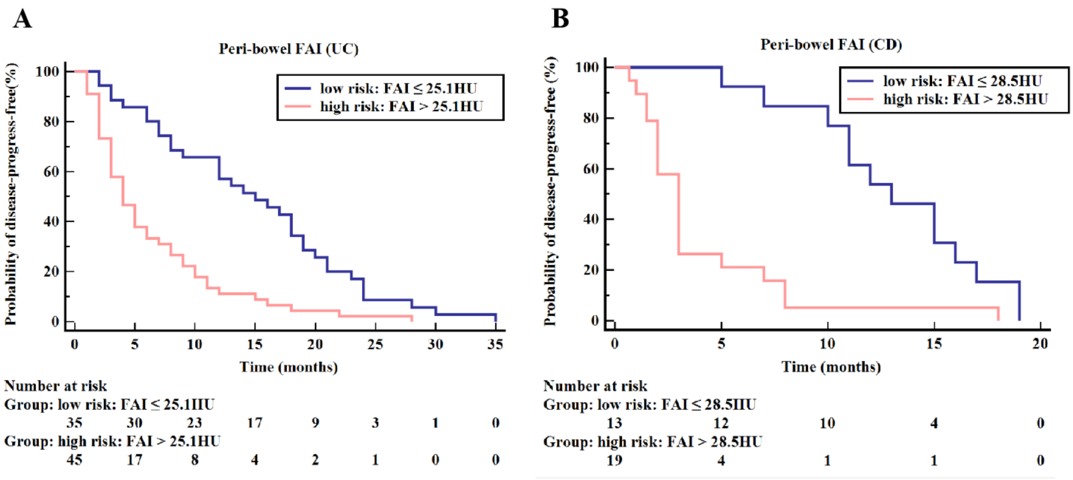


Supplementary Fig. 1 Kaplan-Meier curve for risk of disease progression with high versus low peri-bowel FAI in subgroup analysis. (A) UC cohorts; (B) CD cohorts. FAI, fat attenuation index; UC, ulcerative colitis; CD, Crohn's disease.

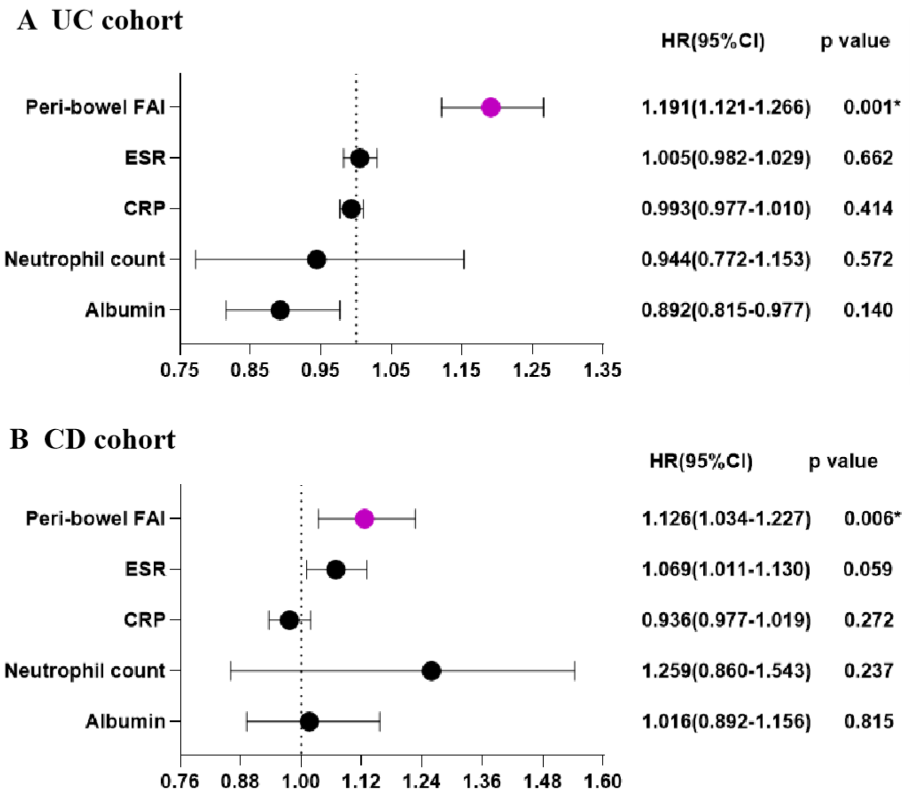


Supplementary Fig. 2 The forest plot of Cox regression analysis with risk factors in subgroup analysis. (A) UC cohorts; (B) CD cohorts. The peri-bowel FAI was an only independent predictor of disease progression in both cohorts. UC, ulcerative colitis; CD, Crohn's disease; FAI, fat attenuation index; ESR, erythrocyte sedimentation rate; CRP, C-reactive protein.

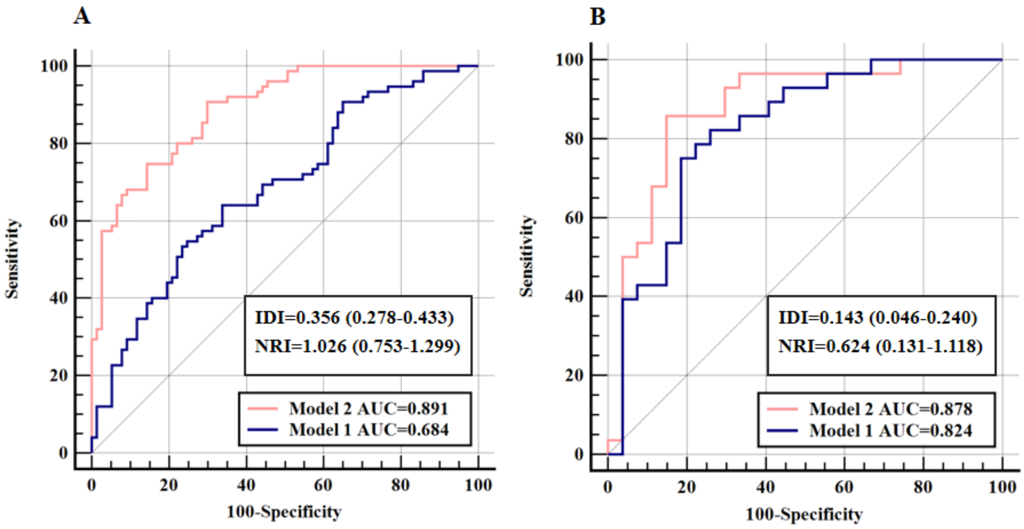


Supplementary Fig. 3 Incremental prognostic value of the peri-bowel FAI for risk stratification. Time-dependent ROC curves (at 18 months) for discrimination and risk classification of disease progression in subgroup analysis. Model 1 represents the current noninvasive risk assessment and consisted of CRP, ESR, neutrophil count and albumin. Model 2 incorporates peri-bowel FAI values into Model 1. (A) UC cohorts; (B) CD cohorts. ROC, receiver operating characteristic; UC, ulcerative colitis; CD, Crohn's disease; CRP, C-reactive protein; ESR, erythrocyte sedimentation rate; IDI, integrated discrimination improvement; NRI, net reclassification improvement.
